# The binding of DCC-P3 motif and FAK-FAT domain mediates the initial step of netrin-1/DCC signaling for axon attraction

**DOI:** 10.1038/s41421-017-0008-8

**Published:** 2018-02-20

**Authors:** Shutong Xu, Yiqiong Liu, Xiaolong Li, Ying Liu, Rob Meijers, Yan Zhang, Jia-huai Wang

**Affiliations:** 1 College of Life Science and Technology, Huazhong Agricultural University, Wuhan, 430070 China; 2 Department of Medical Oncology, and Cancer Biology, Dana-Farber Cancer Institute, Harvard Medical School, Boston, MA 02215 USA; 3 State Key Laboratory of Membrane Biology, College of Life Sciences, Peking University, Beijing, 100871 China; 4 PKU-IDG/McGovern Institute for Brain Research, Peking University, Beijing, 100871 China; 5 European Molecular Biology Laboratory (EMBL), Hamburg Outstation, Notkestrasse 85, D-22607 Hamburg, Germany; 6 Department of Pediatrics, and Biological Chemistry and Molecular Pharmacology, Harvard Medical School, Boston, MA 02215 USA

## Abstract

Netrin-1 plays a key role in axon guidance through binding to its receptor, Deleted in Colorectal Cancer (DCC). The initial step of signaling inside the cell after netrin-1/DCC ligation is the binding of DCC cytoplasmic P3 motif to focal adhesion targeting (FAT) domain of focal adhesion kinase (FAK). Here we report the crystal structure of P3/FAT complex. The helical P3 peptide interacts with a helix-swapped FAT dimer in a 2:2 ratio. Dimeric FAT binding is P3-specific and stabilized by a calcium ion. Biochemical studies showed that DCC-P3 motif and calcium ion could facilitate FAT dimerization in solution. Axon guidance assays confirm that the DCC/FAK complex is essential for netrin-1-induced chemoattraction. We propose that netrin-1/DCC engagement creates a small cluster of P3/FAT for FAK recruitment close to the cell membrane, which exerts a concerted effect with PIP2 for FAK signaling. We also compare P3/FAT binding with paxillin/FAT binding and discuss their distinct recognition specificity on a common FAT domain for axon attraction versus integrin signaling, respectively.

## Introduction

During development, the growth of neuronal axons is guided by combined attractive and repulsive cues in the extracellular environment. Different guidance cue receptors on the growth cone of the axon interact with corresponding guidance cues released from target cells to continually explore the environment. The attractive guidance cues direct axons to their targets, whereas repulsive guidance cues generate exclusion zones that axons avoid, which ensures the correct axon navigation along a defined trajectory out of many possible routes^1,2^. Netrin-1 is the prototypical axon guidance cue characterized in the early 1990s^3,4^. Interestingly, netrin-1 is bi-functional^5,6^. A receptor termed Deleted in Colorectal Cancer (DCC) constitutively expresses on the axonal surface, mediating the chemoattraction by netrin-1^7^. This response is turned into repulsion if another netrin-1 receptor, uncoordinated-5 (UNC5), co-exists with DCC^8^.

The crystal structure of netrin-1 in complex with DCC membrane-proximal fibronectin (FN) type III domains FN5–FN6 reveals that one netrin-1 molecule can simultaneously bind to two DCC receptors through the DCC-specific binding site 1 and a more generic, electrostatic-dominant site 2. Furthermore, we show that UNC5A can functionally replace DCC binding at netrin-1 site 2 to switch the response from attraction to repulsion^9^. Recently, antibody-blocking experiments confirmed the UNC5 binding to site 2 (ref. ^10^). At about the same time in 2014, another structure of the netrin-1 in complex with DCC FN4–FN5 domains was determined^11^. The two netrin-1/DCC complex structures are in fact complementary, as reviewed in ref.^12^.

**Fig. 1:**
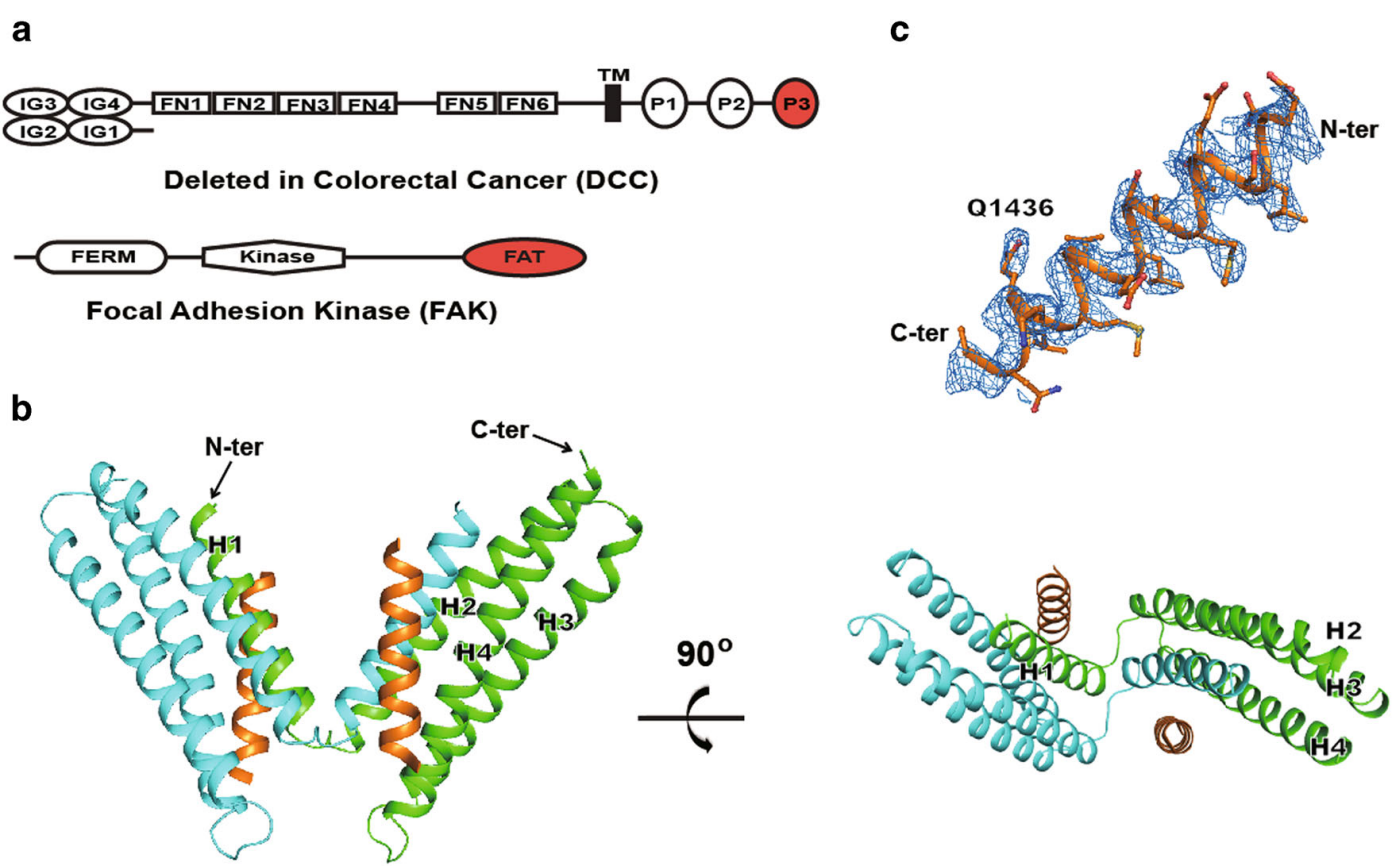
Crystal structure of DCC-P3 motif in complex with FAK-FAT domain. **a** Domain diagram of DCC and FAK, with the region present in the crystal structure in red. **b** Two views of the overall structure of the DCC-P3/FAK-FAT complex. For the helix-swapped FAT dimer, one FAT domain is colored in cyan and the other in green. The two P3 motifs are colored in orange. For one of the FAT domains, the secondary structure elements are marked. **c** A representative simulated-annealing composite omit 2*F*_o_*–F*_c_ electron density map (contoured at 1.0*σ* level) for P3 motif. The Q^1436^ residue that is important for the binding specificity is labeled

The next important question in netrin/receptor signaling concerns the cytoplasmic signaling mechanisms following netrin-1 engagement with DCC on the cell surface. DCC is a single-pass transmembrane receptor composed of 10 extracellular domains and a long cytoplasmic tail of about 350 residues^7^ (Fig. 1a). The cytosolic portion does not appear to intrinsically fold into a defined domain structure. Nevertheless, three highly conserved sequence motifs termed P1, P2, and P3, each consisting of roughly a dozen amino acids, can be identified^13^ (Supplementary Fig. S1). Interestingly, the P3 motif situated at the C-terminal end of DCC’s cytoplasmic tail is shown to be responsible for the netrin-1/DCC-mediated axon attraction^14,15^. The immediate downstream signaling leading to attraction was shown to involve the interaction of the DCC-P3 motif with the focal adhesion kinase (FAK)^16^.

FAK, a non-receptor tyrosine kinase, is composed of an N-terminal 4.1, ezrin, radixin, and moesin homology (FERM) domain, a kinase domain, proline-rich regions, and a C-terminal focal adhesion targeting (FAT) domain (Fig. 1a). FAK plays a major role in transducing signals downstream from membrane receptors, integrins, or growth factor/cytokine receptors^17^. In order for FAK to carry out its function, it has to be localized to focal adhesion site or the cytoplasmic portion of a receptor. For integrin signaling, FAK recruitment is initiated by binding of the FAT domain to paxillin and/or talin while for growth factor/cytokine receptors, FAT binds to their cytoplasmic tails^17^. The FERM domain has also been shown to enhance FAK binding^18,19^. For netrin-1/DCC signaling it has been shown that the FAT domain of FAK is necessary and sufficient for binding to the DCC-P3 motif^16^. FAK, together with Src, can assemble a multi-protein complex to bridge receptor DCC with cytoskeleton-associated proteins, and therefore to regulate neurite outgrowth and growth cone turning. As a central node in the signaling network, FAK must be precisely regulated. At FAK’s resting auto-inhibitory state, the FERM domain directly binds the kinase domain in* cis*, which sequesters auto-phosphorylation site Tyr^397^ located between the FERM and kinase domains from exposure. The FERM-binding also blocks regulatory phosphorylation sites on the activation loop of the kinase domain. FAK activation requires opening the FERM domain from the kinase domain, allowing auto-phosphorylation of Tyr^397^, which becomes a docking site for Src kinase SH2 domain. The Src in turn phosphorylates FAK at the activation loop. The activated FAK/Src complex then further phosphorylates downstream substrates^20^. The central activation issue here is what causes the FERM domain to open up, initiating the process. Very recently, it has been shown that a lipid termed phosphatidylinositol-4,5-bisphosphate, [PI(4,5)P2, or in short PIP2], induces clustering of FAK on the lipid bilayer through binding a conserved basic patch on FERM domain. The neutralization of the basic patch on FERM domain by specific PIP2 binding eventually causes partial domain opening between FERM and kinase, giving rise to FAK activation^21^.

We report here a crystal structure of DCC-P3 motif in complex with FAT domain of FAK. We show that the specific P3-binding facilitates the FAT dimerization through a helix swap mechanism. In addition, FAT dimerization is aided by a calcium ion, which is elevated in cytoplasm via netrin-1 stimulation^22^. Biochemical data revealed that addition of the DCC-P3 motif to FAT results in FAT dimerization in solution. Axon guidance assays confirm that this DCC/FAK complex is physiologically essential for netrin-1-induced chemoattraction. We also show that the P3/FAT interaction is specific for axon attraction signaling and distinct from FAT bound by LD motifs from the paxillin family for integrin focal adhesion signaling. We propose that ligation of netrin-1 with DCC at the cell surface creates a small cluster of P3/FAT for FAK recruitment to proximity of the cell membrane, which exerts a concerted effect with PIP2 for FAK signaling. To our knowledge, this has been the first structural observation of the initial signaling step after ligand/receptor engagement on the cell surface for the axon guidance process.

## Results

### Netrin-1 engagement facilitates P3-23m peptide binding to a helix-swapped FAT dimer

Previous biochemical data have shown that deletion of residues S^1424^–S^1443^ within the P3 motif completely abolishes the binding between DCC and FAK, defining this peptide segment as the region required for the FAK binding^16,23^. On the FAK side, its C-terminal FAT domain is responsible for the DCC binding^16^ (Fig. 1a). To structurally characterize the DCC/FAK binding, a 23-mer peptide encompassing residues D^1421^–S^1443^ from the rat DCC-P3 motif, designated as P3-23m, and a FAT construct encompassing N^921^-L^1046^ from mouse FAK were used in this study.

**Fig. 2:**
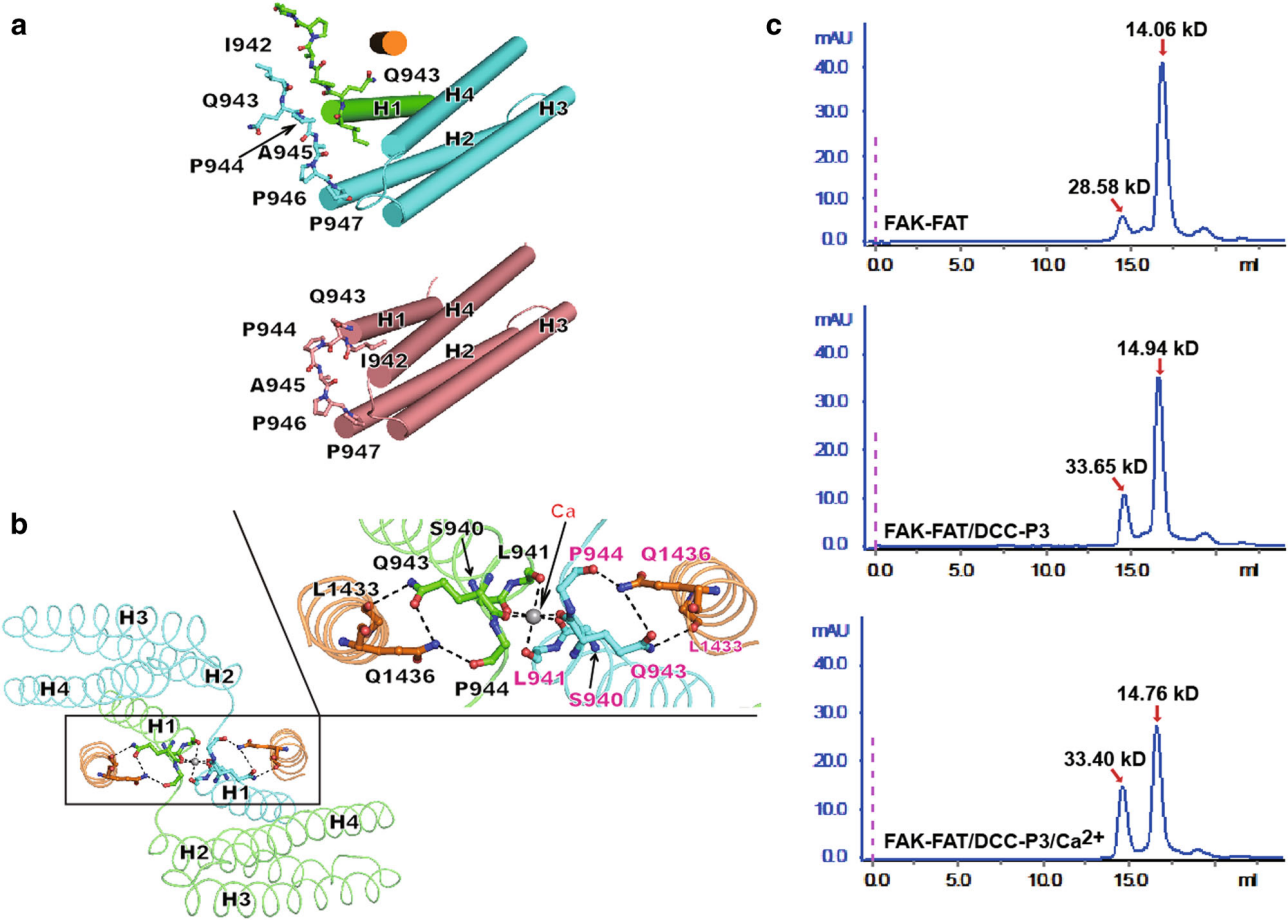
Characteristic features of the P3-bound FAT dimer. **a** Comparison of the FAT 4-helix bundle structures in monomer and dimer. Upper and lower panels show the 4-helix bundles in the 2:2 P3/FAT dimer and the FAT monomer (apo-form) (PDB code 1K40), respectively. Residues on the H1/H2 loop are shown with ball-and-stick models and labeled. The secondary structure elements of the FAT domains are labeled. The color coding for the 2:2 P3/FAT dimer is the same as in Fig. 1b. The apo-FAT monomer is colored in salmon. **b** Dimer interface in the P3/FAT complex. In the left panel, the secondary structure elements of the FAT domains are labeled. Right panel is a close-up view of the dimer interface. The hydrogen bonds and metal ion coordination bonds are indicated with black dashed lines. The calcium ion is shown in gray sphere. Side-chain or main-chain atoms of the residues involved in stabilizing the dimer are shown with ball-and-stick models. For clarity, residues from one P3/FAT protomer are labeled in black and the other in magenta. The color coding for the P3/FAT complex is the same as in Fig. 1b. **c** SEC-MALS assay showed that addition of the DCC-P3 peptide and Ca^2+^ to FAT results in FAT dimerization in solution. Upper panel: The elution profile of FAT alone. Middle panel: The elution profile of FAT in the presence of DCC-P3 peptide. Lower panel: The elution profile of FAT in the presence of DCC-P3 peptide and Ca^2+^

The P3/FAT complex structure, which was refined to 2.5 Å resolution (Table 1), assumes a butterfly shape with P3-23m bound to FAT in a 2:2 dimeric form (Fig. 1b). A monomeric form of the apo-FAT structure, first published in 2002, adopts an up-and-down 4-helix bundle (PDB code 1K40)^24^. The most interesting feature of the P3-23m/FAT complex structure is that the N-terminal helix 1 (H1) of the FAT domain swaps between the two protomers. The P3-23m peptide folds into a helix, packing onto the H1/H4 face of the FAT domain with well-defined electron density (Fig. 1c). Remarkably, the swapped H1 packs onto the H2–H3–H4 in our structure essentially in the same fashion as does H1 within the monomeric form of apo-FAT. Each of the two 4-helix bundles of our dimeric structure significantly resembles the bundle in the monomeric structure (PDB code 1K40) with the RMSD value of only 1.18 and 1.36 Å (superposition of Cα atoms), respectively (Fig. 2a). Apparently, the extensive hydrophobic side-chain interactions among the helices inside the bundle make the 4-helix bundle quite robust, be it a monomer or a swapped dimer. On the contrary, the H1–H2 loops assume different conformations in the FAT dimer versus monomer (Fig. 2a).

**Table 1:** Summary of diffraction data and structure refinement statistics

Diffraction data	
Wavelength (Å)	0.979
Space group	C2
Cell parameters	
*a*, *b*, *c* (Å)	129.2, 36.1, 65.4
*α*, *β*, *γ* (°)	90.0, 104.3, 90.0
Resolution (Å)	50–2.5 (2.59–2.50)^a^
Observed reflections	33,829
Unique reflections (I/*σ*(I) > 0)	10,318
Average redundancy	3.3 (3.1)
Average I/*σ*(I)	12.2 (2.0)
Completeness (%)	98.7 (93.4)
*R*_merge_ (%)^b^	9.2 (52.7)
Refinement and structure model	
Reflections (*F*_o_ ≥ 0*σ*(*F*_o_))	
Working set	10,314
Test set	496
*R* factor/Free *R* factor (%)^c^	22.6/26.9
Average *B* factor (Å^2^)	
All atoms	64.3
Protein (FAT)	62.4
Ion	81.5
Peptide (P3)	78.1
Water	53.8
RMS deviations	
Bond lengths (Å)	0.006
Bond angles (°)	0.573
Ramachandran plot (%)	
Favored	99.3
Allowed	100

^a^ Numbers in parentheses represent the highest resolution shell

^b^ *R*_merge_ = ∑_*hkl*_∑_*i*_|*I*_*i*_(*hkl*)_*i*_ − <*I*(*hkl*)>|/∑_*hkl*_∑_*i*_*I*_*i*_(*hkl*)

^c^ *R* = ∑_*hkl*_||*F*_o_| − |*F*_c_||/∑_*hkl*_|*F*_o_|

Deposited in the Protein Data Bank are 23 FAT domain structures, containing more than three dozen independent FAT molecules, unligated or ligated, determined by x-ray crystallography or NMR (Supplementary Table S1). The overwhelming majority is monomeric while only two are dimeric. Arold and his colleagues first observed an H1-swapped dimer of apo-FAT domain. It took 3 months for the crystals to appear; another 3 months to grow big enough for diffraction-data collection. They were unable to reproduce the crystals^25^. Another H1-swapped dimer was observed in the structure of FAT in complex with the paxillin LD4 motif. In that crystal structure, three independent FAT molecules exist in one asymmetric unit. Interestingly, one FAT monomer binds one paxillin, while another monomer has no ligand binding, and only the third FAT molecule pairs with a symmetry-related molecule to form an H1-swapped dimer to bind paxillin^26^. The H1-swapped dimer appears to represent a very minor population of FAT domains in dynamic equilibrium. Using a native-state hydrogen exchange technique, a hidden folding intermediate of FAT was directly detected, in which the N-terminal helix H1 was unfolded while the rest H2–H3–H4 body remained folded. Under the conditions of the experiments, this H1-unfolded intermediate was an extremely small population^27^. The conformational dynamics of H1 opening seems an essential regulator of FAK cellular function. More importantly, mutagenesis and biochemical studies suggest that the opening of H1 is likely owing to the strain introduced by the short proline-rich H1–H2 loop of I^942^QPAPP^28^.

Contrary to above observations, our P3/FAT structure is in a stable H1-swapped dimeric form, easy to co-crystallize. Two notable structural features appear to stabilize the swapping (Fig. 2b). First, in the swapped dimer, the two H1–H2 loops are close to each other with a calcium ion sitting right between them. The ion is coordinated with six main-chain carbonyl oxygen atoms, three from each protomer, in a perfect octahedral geometry. The ion coordination bond lengths are all around 2.3–2.6 Å, chemically suggesting that it should be a calcium ion^29^. Secondary, there are several hydrogen bonds in each protomer between the P3-23m and FAT domain. The side chain of P3-23m Q^1436^ is sandwiched between the main-chain carbonyl oxygen of FAT P^944^ and the side chain of FAT Q^943^. The main-chain carbonyl oxygen of P3-23m L^1433^ forms another hydrogen bond with the side chain of FAT Q^943^. Remarkably, these hydrogen bonds can only take place when the FAT domain is in the H1-swapped dimeric form but not in the monomeric form (Fig. 2a, b).

The SEC-MALS analyses showed that FAT alone mainly forms monomer in solution (Fig. 2c). In the presence of the DCC-P3 motif and Ca^2+^, the amount of FAT dimers apparently increased (Fig. 2c). In addition, compared to FAT dimers, the M.W. (molecular weight) of ligand-bound FAT dimers is about 5 kDa heavier (Fig. 2c). The theoretical M.W. of DCC-P3 motif is 2.45 kDa. These results strongly indicated that DCC-P3 motif was bound with the FAT in 2:2 form in solution, which is perfectly consistent with our 2:2 P3/FAT structure. Taken together, the structural and biochemical data demonstrated that DCC-P3 motif and Ca^2+^ could facilitate the dimerization of FAT.

### The FAT-binding specificity of P3 versus LD motif for differential signaling

Acting as a protein–protein interacting module, the FAT domain has multiple binding partners to help FAK localize and function in an appropriate cellular context^19^. The two hydrophobic patches on the opposite faces of the FAT 4-helix bundle structure, termed as H1/H4 face and H2/H3 face, are predicted to be ideal for partner binding^24^. The most extensively studied ligand for FAT is paxillin, through which numerous cellular signaling pathways, in particular integrin-associated ones, get coordinated at the focal adhesion site. Paxillin consists of five protein-binding LD motifs, named after two strictly conserved leucine (L) and aspartate (D) residues. Although these LD motifs have a consensus sequence, LDxLLxxL, their interactions with multiple proteins seem both overlapping and specific^30^. Only the LD2 and LD4 motifs of paxillin are able to bind the FAK-FAT domain^31^. Proline-rich tyrosine kinase 2 (Pyk2), which is a more tissue-specific FAK family member, also has a C-terminal FAT domain that binds leupaxin, a leukocyte-specific isoform of paxillin. Leupaxin has four LD motifs, of which only LD1 and LD4 bind FAT^32^. Leupaxin is regarded as Pky2’s native ligand, but intriguingly paxillin LD motifs can also bind Pky2-FAT domain^32,33^. As a versatile protein-binding module with a shared binding mode for various ligands, it is biologically important that the FAT domain should also possess binding specificity for different ligands.

**Fig. 3:**
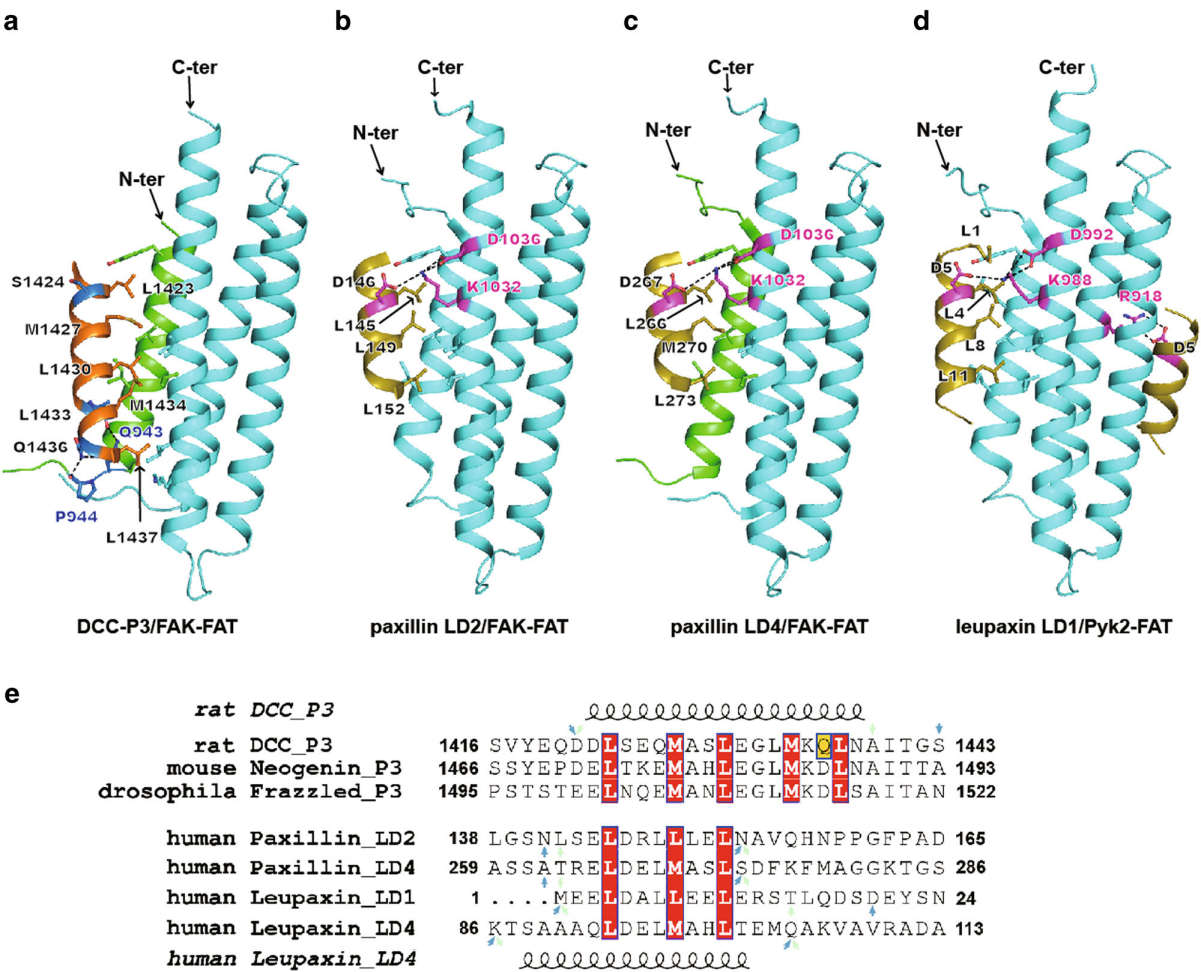
Comparison of the P3/FAT complex with LD/FAT complexes. The crystal structures of DCC-P3/FAK-FAT (**a**), paxillin LD2/FAK-FAT (PDB code 1OW8) (**b**), paxillin LD4/FAK-FAT (PDB code 1OW6) (**c**), and leupaxin LD1/Pyk2-FAT (PDB code 4XEF) (**d**) are shown in the same orientation. The hydrogen bonds are indicated with black dashed lines. Side-chain or main-chain atoms of the residues involved in binding are shown with ball-and-stick models as is the side chain of DCC-P3 S^1424^ that is equivalent to the invariant aspartate residues in the LD motifs. At the H2/H3 interface of the leupaxin LD1/Pyk2-FAT complex, side chains of leupaxin LD1 D^5^ and Pyk2-FAT R^918^ are also shown as ball-and-stick models. Residues of P3 and LD motifs are labeled in black. Concerning the FAT domains, residues involved in hydrogen-bond interactions are labeled in marine for the P3/FAT complex and magenta for LD/FAT complexes. For FAT, the H2–H3–H4 body is colored in cyan while the H1 is colored in green in the dimeric form and in cyan in the monomeric form. The DCC-P3 motif and the LD motifs are colored in orange and olive, respectively. **e** Structure-based sequence alignment of P3 motifs of rat DCC and its homologs (mouse Neogenin and *Drosophila* Frazzled) and LD motifs of human paxillin and human leupaxin. The hydrophobic residues at the ‘a’ or ‘d’ positions of heptad repeats are shaded in red. The residue Q^1436^, important for P3 and FAT-binding specificity, is in yellow. The resolved regions of the LD peptides in the crystal structures of paxillin LD2/FAK-FAT (PDB code 1OW8), paxillin LD4/FAK-FAT (PDB code 1OW6), leupaxin LD1/Pyk2-FAT (PDB code 4XEF), and leupaxin LD4/Pyk2-FAT (PDB code 4XEV) are indicated with cyan arrows. The starting and ending residues of the peptides used for crystallization are indicated with magenta arrows. The sequence numbers of each motif and the secondary structure elements of DCC-P3 motif and leupaxin LD4 motif are marked

So far, several structures of the FAT domain in complex with LD motif have been determined (Supplementary Table S1). To explore the specific recognition for DCC axon guidance signaling as opposed to integrin focal adhesion signaling, we compared the structures of P3/FAT complex with LD/FAT complexes (Fig. 3a–d and Supplementary Fig. S2). All of the FAT-binding motifs share one feature in common, namely to employ the central hydrophobic face, with leucine or methionine, of their amphipathic helices for the interface contact. The LD motifs are found to bind at both the H1/H4 and H2/H3 faces. Yet, structural observations and biochemical data indicate that the H2/H3 face is a stronger binding site for the leupaxin LD4 motif^32^. By contrast DCC-P3 only binds the H1/H4 face on a dimeric form of FAT domain.

Although both P3 and LD motifs assume helical conformations, compared with LD motifs, the P3 motif extends two more helical turns at the C-terminus with two additional hydrophobic residues, L^1433^ and L^1437^, in contact with the FAT domain (Fig. 3a–d). Structure-based sequence alignment of P3 motifs from DCC and its homologs with LD motifs from paxillin and leupaxin reveals one more Leu-zipper style heptad-repeat contained in the P3 motifs than is contained in any LD motif (Fig. 3e). It suggests that the residues following LD motifs (ending at the magenta colored arrow in Fig. 3e) could not adopt a P3-like extended amphipathic helix for interaction with the FAT domain. As discussed above, P3/FAT binding is sequence-specific and Ca^2+^-dependent. Within the P3/FAT complex, two glutamine residues, Q^943^ of FAT and Q^1436^ of P3, play a key role in FAT binding at the H1–H2 loop (Figs. 2b and 3a). The residue Q^1436^ is located near the C-terminus of P3’s elongated helix. It is clear that none of the known LD motifs has a defined structure able to reach as far as Q^1436^ of P3 for partner binding. This suggests that Q^943^Q^1436^-mediated FAT binding is P3-specific.

The LD/FAT binding is also specific. The characteristic feature of LD motifs is an invariant aspartate residue following a leucine^34^. This aspartate residue (D^146^ in paxillin LD2, D^267^ in paxillin LD4, D^5^ in leupaxin LD1, and D^94^ in leupaxin LD4) plays a very important role in electrostatic interactions with the FAT domain. On the H1/H4 face, the conserved aspartate residue forms hydrogen bonds with a lysine residue on the H4 helix of the FAT domain (K^1032^ in FAK-FAT and K^988^ in Pyk2-FAT). Interestingly, this lysine residue is also stabilized by another aspartate residue on the same H4 helix (D^1036^ in FAK-FAT and D^992^ in Pyk2-FAT) (Fig. 3b–d). On the H2/H3 face, the conserved aspartate residue forms a bidentate hydrogen bond with an arginine residue on the H2 helix of the FAT domain (R^962^ in FAK-FAT and R^918^ in Pyk2-FAT) (Fig. 3d and Supplementary Fig. S2). A systematic survey on all published LD/FAT complex structures demonstrates that the D-K-D sandwich pattern for LD binding at the H1/H4 face and the D–R interaction at the H2/H3 face of FAT domain are structurally conserved. In the DCC-P3 motif, the equivalent residue of this conserved aspartate residue is S^1424^, whose side chain is shorter and does not form any contact with the FAT domain (Fig. 3a). In other words, the invariant aspartate residue near the N-terminus of the LD motif provides register specificity on FAT binding, which is not present in the P3 motif for FAT binding.

In summary, the 4-helix bundle FAT domain with two hydrophobic binding faces serves as a versatile protein–protein interacting module for many ligands. On the other hand, exquisite and differential structural features have defined recognition specificity for different ligands. While the LD-mediated electrostatic LD/FAT interaction specifies integrin-associated focal adhesion signaling, the QQ-mediated specific dimeric P3/FAT interaction appears unique for axon attraction signaling triggered by netrin-1/DCC engagement.

We should mention here that DCC-P3 was also reported to bind the MyTH4-FERM domain of an unconventional myosin family member, Myosin X, as described in a rat DCC-P3/MyTH4-FERM complex structure^35^. The P3 motif also assumes a helical conformation. This P3 helix binds to a αβ-groove of the FERM-F3 lobe (Supplementary Fig. S3). This α-helix (α1) in MyTH4-FERM domains is in a similar position as H4 in the FAT domain but much shorter, whereas the short β-strand (β5) in MyTH4-FERM domain contacts a distinct region of rat DCC-P3. Interestingly, similar to our DCC-P3/FAT complex, the Q^1436^ of DCC-P3 also forms two hydrogen bonds with the main chains of S^2001^ and F^2002^ on MyTH4-FERM β5, possibly contributing to the recognition specificity as well.

### Axon guidance assay confirms that the DCC/FAK complex is essential for netrin-1 induced chemoattraction

In the crystal structure of DCC-P3/FAK-FAT complex, hydrophobic residues including L^1423^, M^1427^, L^1430^, L^1433^, M^1434^, and L^1437^ are clustered on the one side of the P3 motif and form strong hydrophobic interaction with FAK-FAT domain. Besides, P3 Q^1436^ and L^1433^ form hydrogen bonds with FAT Q^943^ and P^944^ (Fig. 3a). The P3 domains in DCC and its homolog Neogenin^36^ shares 71% identity with those key hydrophobic residues strictly conserved (Fig. 3e). The equivalent residues of rat DCC L^1430^/L^1433^/L^1437^ are L^1480^/L^1483^/L^1487^ in mouse Neogenin, respectively. Previous yeast-two-hybrid assays showed that wild-type Neogenin can interact with FAK and mutation of any one of the three leucine residues completely abolished the Neogenin/FAK binding^16^. These results suggest that mutation of DCC L^1430^/L^1433^/L^1437^ could also abrogate the DCC/FAK interaction, which is excellently consistent with our structural observations.

**Fig. 4:**
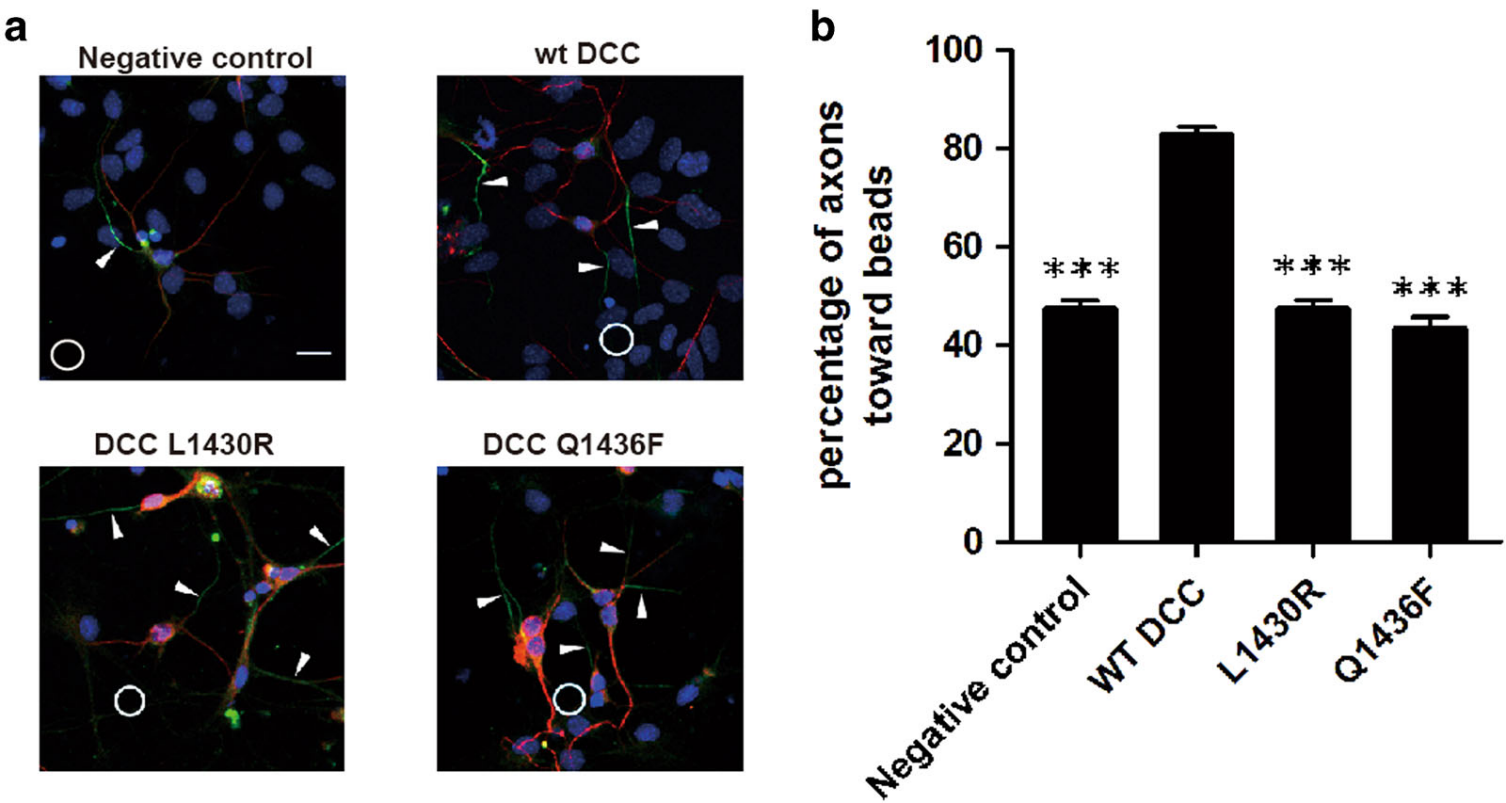
Axon guidance assay verifying the importance of DCC-P3/FAK-FAT binding in netrin-1-induced axon attraction. **a** Representative images of axon guidance assays for WT and mutant DCC affecting DCC-P3/FAK-FAT binding. Phosphate-buffer saline was used as a negative control. The position of the bead coated with netrin-1 is marked with a circle. The injected cells are indicated by DTR (red). Cell bodies are dyed with DAPI (blue). Growing axons are marked with anti-AnkG antibody (green) and indicated by white arrows. The scale bar denotes 10 μm. **b** Statistics data showing the percentage of axons attracted by the beads coated with netrin-1. DCC mutants affecting DCC-FAK binding abolish attraction. Data represent mean ± SE (*n* = 50 for each group). One-way ANOVA followed by a post hoc Scheffe´’s test were performed. **p < 0.01 compared with wild type

To study the effect of changes in the DCC/FAK binding in vivo, we performed axon guidance assays on neurons harvested from the hippocampus of C57BL/6 mice. Individual neurons were cultured on a Petri dish with a netrin-1-soaked bead at one corner. The endogenous DCC gene within the neurons was knocked down using siRNA. The ability to rescue chemoattraction by microinjection with a vector containing wild type or mutant DCC was checked (Fig. 4a and Supplementary Fig. S4A). Phosphate-buffer saline (PBS) was used as a negative control. For neurons microinjected with PBS, the axons were grown randomly with about 50% of them towards netrin-1 and the other 50% far away from netrin-1. For neurons microinjected with wild-type DCC, more than 80% of the axons were grown towards netrin-1, indicating an axon attraction effect (Fig. 4b and Supplementary Fig. S4B). Mutation of DCC L^1430^ to Arg impaired the hydrophobic interaction between DCC and FAK while mutation of DCC Q^1436^ to Phe disrupted its hydrogen-bond interaction with FAT Q^943^ and P^944^, both leading to lose FAK binding. For neurons microinjected with either of the two DCC mutants, the axon grown towards netrin-1 was reduced to 40–50%, indicating the two DCC mutants totally abolished the axon attraction effect. These data demonstrated that impairing the DCC/FAK interaction would affect netrin-1 induced axon attraction. In other words, the axon guidance assays shown here demonstrate that the DCC/FAK interaction we observed in the structure is of biological significance for netrin-1-induced chemoattraction.

## Discussion

### The H1-swapped dimeric FAT domain in complex with P3-23m is of biological significance

As demonstrated by the previous structural and biochemical data, one netrin-1 brings two DCC receptors in close proximity on the membrane of the growth cone of developing axons^9^. This creates an opportunity for two P3 motifs in the DCC cytoplasmic tails to specifically ligate FAT domain of FAK into an H1-swapped dimeric form as described above. Netrin-1, through its activation of DCC, also causes significant membrane depolarization, which triggers elevation of the cytosolic Ca^2+^ level^22^. Netrin-1-induced Ca^2+^ influx was shown to be required for the turning of growth cones of cultured *Xenopus* spinal neurons^37^. Hence it is conceivable that the calcium ion observed in our crystal structure is physiological. Elevated Ca^2+^ should facilitate in vivo dimeric DCC-P3/FAK-FAT complex formation upon netrin-1 engagement. In sum, the FAT domain is inherently in dynamic equilibrium with a tendency to have its H1 helix swing out to form a dimer. Netrin-1 triggered DCC signaling facilitates the P3 motif to bind a swapped FAT, forming a stable dimer with the help of Ca^2+^, which initiates the ensuing process of intracellular signal transduction for axon attraction.

### A model of P3/FAT interaction triggered DCC signaling

Signal transduction through transmembrane receptors is one of the central issues in biology. There have been numerous studies on how a ligand binds to its cognate receptor on the cell surface as well as how the signal is transduced inside the cell. The most elusive aspects are the mechanisms which couple the events exterior to the plasma membrane to the biochemical events identified in the cytoplasm. The P3/FAT structure presented here represents the first description of a mechanism providing the initial step of axon attraction signaling inside the cell triggered by netrin-1/DCC engagement on the cell surface.

**Fig. 5:**
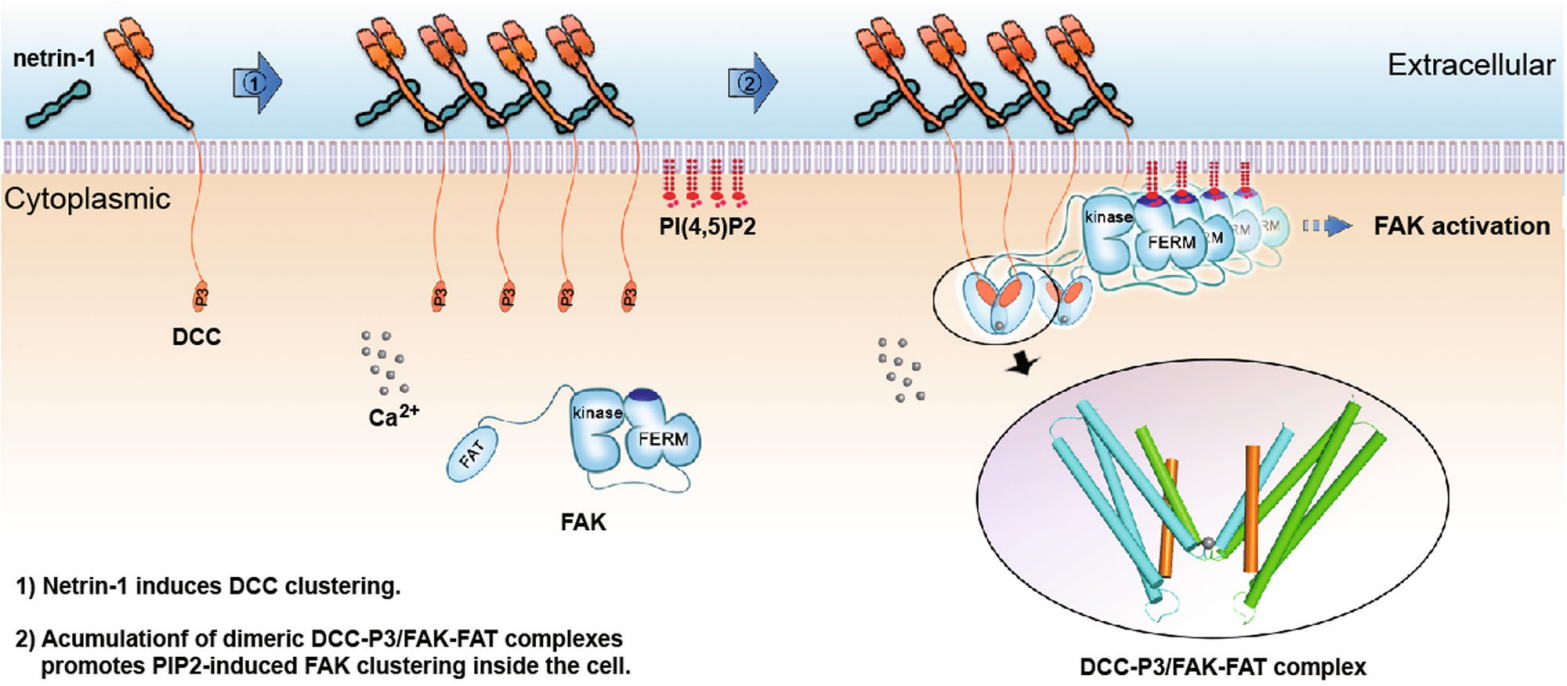
A model for netrin-1/DCC engagement leading to intracellular FAK/Src signaling via DCC-P3 and FAK-FAT binding (proposed signaling sequence step-wise from left to right). DCC is expressed on the growth cone of the axon. (Step 1) Netrin-1 binds to DCC to cause DCC clustering. (Step2) Two P3 motifs in the DCC cytoplasmic tails specifically ligate FAT domain of FAK into H1-swapped dimeric form, which is further stabilized by netrin-1 induced Ca^2+^ influx. The small cluster of netrin-1/DCC pairs on the cell surface puts P3-facilitated FAK dimers in proximity, resulting in a local accumulation of FAK dimers, promoting PIP2-induced FAK clustering. This eventually gives rise to FAK activation, a process initiated by PIP2 to release auto-inhibition of the FAK kinase domain by the FAK FERM domain^21^

Our findings together with a number of previous reports suggest a rather complex model of how P3/FAT binding leads to FAK/Src signaling, as presented in Fig. 5. Our netrin-1/DCC FN5–FN6 structure showed that one netrin-1 ligand can bring together two DCC receptors^9^. Complemented by the netrin-1/DCC FN4–FN5 structure, it appears that upon netrin-1 engagement, DCC could form a small cluster on the surface of the axon growth cone resulting in axon attraction^12^ (Supplementary Fig. S5). This cluster is not linear zipper-like, but two-dimensional. Ligand-induced receptor clustering always plays a critical role in signal transduction^38^. In the case of netrin-1-induced DCC clustering, less than 5 netrin-1 molecules seem sufficient to initiate axon attraction^39^. The P3/FAT structure demonstrates that the cytoplasmic tail of DCC homo-dimerizes through its P3 motif, engaging with the helix-swapped FAT dimer, which could be further stabilized by netrin-1-induced Ca^2+^ influx. The cluster of netrin-1/DCC pairs on the cell surface creates an opportunity for P3-facilitated FAK dimers to be close to one another, resulting in local accumulation of FAK dimers, in favor of PIP2-induced FAK clustering inside the cell. This eventually gives rise to FAK activation, a process that is initiated by PIP2 to release auto-inhibition of the kinase domain by the FERM domain^21^. The neutralization of the enriched acidic PIP2 on the plasma membrane with a positive patch on the FERM surface is shown to be a critical factor. Interestingly, the membrane-proximal region of DCC cytoplasmic tail is also rich in basic residues (Supplementary Fig. S1). This may help consolidate the clustering near the membrane for kinase function. Moreover, as mentioned above, the long DCC cytoplasmic tail is intrinsically disordered. It contains as much as 15% prolines as opposed to around 5% in normal proteins^40^. This should make the whole cytoplasmic tail stiffer and more extended. It helps the C-terminal P3 motif to reach out from membrane inside the cell for the recruitment of FAK. N-terminal to the P3 motif, there is a P^1400^ATP Src-SH3-binding site and Y^1420^ as the major FAK-dependent Src phosphorylation site^41^ (Supplementary Fig. S1). These should all enhance the signal transduction cascade after the initial step of P3/FAT binding.

It should be emphasized that netrin-1 failed to increase FAK tyrosine phosphorylation in cells expressing UNC5B^16^. There is good evidence indicating that the binding of netrin-1 to DCC and UNC5 will turn the attraction into repulsion^9,14^. The signaling of axon repulsion must employ a very different mechanism.

## Material and methods

### Protein expression and purification

FAT encoding N^921^-L^1046^ was amplified from mouse FAK gene contained in pRKVSV vector (Addgene, plasmid #50533). The fragment was inserted into the vector pET-Sumo vector with a 6His and sumo tag at the N-terminus. The plasmid was transformed into *Escherichia coli* BL 21(DE3) (Novagen). The transformed bacteria were cultivated in LB with kanamycin selection. Recombinant protein synthesis was induced when the bacterial culture reached an optical density at 600 nm of 0.8–1.0 by addition of isopropyl β-d-thiogalactopyranoside (IPTG) to a final concentration of 0.2 mM. After 20 h of cultivation at 20 °C, bacteria from 1 l of culture were harvested by centrifugation. Frozen cells were resuspended in 10 vol of lysis buffer containing 50 mM Tris pH 8.0, 300 mM NaCl, 10% (vol/vol) glycerol, and 1 mM phenylmethane sulfonyl fluoride (PMSF), followed by ultrasonication. The lysate was cleared by centrifugation at 16,000 *g* for 20 min and incubated with Ni-NTA resin (Ni-NTA Superflow, Qiagen). The resin was washed with wash buffer containing 20 mM Tris pH 8.0, 100 mM NaCl, 1 mM PMSF, and 20 mM Imidazole. Target protein was eluted with buffer containing 20 mM Tris pH 8.0, 100 mM NaCl, 1 mM PMSF, and 200 mM Imidazole. Target protein was then mixed up with Ulp1 protease at a ratio of 125:1. The mixture was incubated at 4 °C for overnight while dialysis with buffer containing 20 mM Tris pH 8.0, 100 mM NaCl, and 1 mM PMSF. Then the sumo and the uncleaved protein with sumo tag were removed by a second step of His affinity purification. Target protein without tag was further purified by size exclusion chromatography on a Superdex 200 10/300 column (GE Healthcare) in 20 mM Tris pH 8.0 and 100 mM NaCl. Fractions containing the monomer FAT domain were pooled and concentrated.

### Crystallization

The synthetic DCC-P3-23m peptide (DDLSEQMASLEGLMKQLNAITGS) was dissolved with dimethyl sulfoxide. Fresh purified FAT domain was then mixed up with the DCC-P3-23m peptide at a molar ratio of 1:4 and diluted to a final concentration of 25 mg/ml. The mixture was then subject to crystallization using hanging drop vapor diffusion method at 16 °C.

Crystals of FAT domain in complex with DCC-P3-23m peptide appeared in 3 days and were harvested after 6 days. One microliter of protein solution was mixed with 1 μl of crystallization solution containing 20% PEG8000 and 0.2 M MgCl_2_ in 0.1 M Tris buffer (pH 8.5). Before diffraction experiments, crystals were soaked in crystallization solution containing additional 10% glycerol for cryoprotection.

### Structure determination

The diffraction data were collected at Advanced Photon Source (APS) 19 ID. The data were processed, integrated, and scaled together with HKL3000 (ref. ^42^). The statistics of the diffraction data are summarized in Table 1. The structure of FAT in complex with DCC-P3-23m was solved by the molecular replacement method using Phaser^43^ with the structure of apo FAT as the search model (PDB code 1K40). The model building was performed using Coot^44^, and the structure refinement was carried out using Phenix^45,46^. The stereochemical geometry of the structures was analyzed using Molprobity^47^. The figures were generated using Pymol (http://www.pymol.org). The statistics of the structure refinement are also summarized in Table 1. The structure has been deposited in PDB with the code of 6BZ3.

### SEC-MALS assay

A Superdex 200 increase 10/300 column (GE Healthcare) was used in this experiment. For FAT alone, 100 μg FAT was loaded into the column and analyzed in the buffer containing 20 mM HEPES pH 7.4, 150 mM NaCl, and 10 mM EGTA. To test whether DCC-P3 peptide could facilitate FAT dimerization, 100 μg FAT was mixed with DCC-P3 peptide at a molar ratio of 1:4 and then analyzed in the buffer containing 20 mM HEPES pH 7.4, 150 mM NaCl, and 10 mM EGTA. To further test whether Ca^2+^ could facilitate FAT dimerization, 100 μg FAT was mixed with DCC-P3 peptide at a molar ratio of 1:4 and then analyzed in the buffer containing 20 mM HEPES pH 7.4, 150 mM NaCl, and 10 mM CaCl_2_.

### Axon guidance assay

Primary neurons were cultured from newborn C57BL/6 mouse hippocampus^48^ following the regulations of the Peking University Animal Care and User Committee. Axon guidance assays were performed as previously described^9^ with slight modification. At 1 day in vivo, the mouse neurons were microinjected with siRNA to DCC (Qiagen), wild-type DCC/mutant DCC constructs (rat DCC in a pCDNA3.1 vector) with the marker dye dextran Texas Red (DTR, Molecular Probes). The heparin pre-coated beads (Sigma-Aldrich) were then coated with 100 µg/ml netrin-1, dipped with 0.1% agarose (Sigma), and then placed at one corner of the culture dish. After 72 h incubation, neurons were fixed and immune-stained with anti-AnkG antibody (green) (Life Technologies). The direction of axon (green fluorescent) growth of the injected cells (red fluorescent) toward the beads was observed. Axons were considered to be attracted by beads if they pointed toward a plane that lies perpendicular to the axis drawn between the nucleus of the neuron and the center of the bead. A Zesis LSM-710 laser confocal microscope was used for analyzing data. Each image was collected using a ×40 water immersion objective.

## Electronic supplementary material

Supplementary Information
