# Supplementary material for: The binding of DCC-P3 motif and FAK-FAT domain mediates the initial step of netrin-1/DCC signaling for axon attraction
Source: Cell Discov. 2018 Feb 20;4:8. doi: 10.1038/s41421-017-0008-8 (PMC5818605; doi:10.1038/s41421-017-0008-8)
Supplement: Supplementary file 1 — Supplementary Information [file 41421_2017_8_MOESM1_ESM.pdf]

Supplemental figure legend

Fig. S1. Sequence alignment of the cytoplasmic tails in human DCC and its homologs, human Neogenin and *Drosophila* Frazzled. The P1, P2 and P3 motifs and the basic region are labeled. Residues of the P1, P2 and P3 motifs, the proline residues and the positive-charged residues in the basic region of DCC cytoplasmic tail are colored in orange, marine and magenta, respectively. The sequence numbers are marked.

Fig. S2. Comparison of the H2/H3 interfaces within LD/FAT complexes. The crystal structures of paxillin LD2/FAK-FAT (PDB code 1OW8) (A), paxillin LD4/FAK-FAT (PDB code 1OW7) (B), and leupaxin LD1/Pyk2-FAT (PDB code 4XEF) (C), and leupaxin LD4/Pyk2-FAT (PDB code 4XEK) (D) are shown in the same orientation. The hydrogen bonds are indicated with black dashed lines. For LD motifs (olive), residues involved in binding are shown with ball-and-stick models and labeled in black. For FAT domains (cyan), residues involved in binding are shown with ball-and-stick models and those involved in hydrogen-bond interactions are labeled in magenta.

Fig. S3. Comparison of DCC-P3/FAK-FAT and DCC-P3/ Myosin X MyTH4-FERM complexes. The hydrogen bonds are indicated with black dashed lines. The side chains of the residues involved in binding are shown with ball-and-stick models. Residues of P3 motifs are labeled in black. For the FAT and MyTH4-FERM domains, residues involved in hydrogen-bond interactions are labeled in magenta. The color coding for P3/FAT complex is the same as in Figure 1B. In the P3/MyTH4-FERM complex, DCC P3 motif, MyTH4-FERM domains and the linker between them are colored in orange, yellow and gray, respectively.

Fig. S4. Axon guidance assay showing that DCC mutants affecting DCC-FAK binding abolish attraction. (A) As shown in the cartoon model, the angle α was measured for each injected neuron. If the angle is less than 90°, the effect was termed as attraction. If the angle is larger than

90°, the effect was termed as repulsion. (B) Statistics data showing the distribution of angle α. DCC mutants affecting DCC-FAK binding abolish attraction.

Fig. S5. A stereo view of the netrin-1/DCC cluster. The upper panel is a diagram of DCC and Netrin-1 domains. In the netrin-1/DCC cluster (lower panel), the netrin-1s are colored in cyan and green while the DCCs are colored in red and magenta. The domains in netrin-1 and DCC are labeled.

Table S1. Statistics of the FAT-related structures in Protein Data Bank

Supplemental figures

Figure S1

```
                 |-------- Basic region --------||-------P1--------|
Hs_DCC         CTRRSSAQQRKKRATHS----AGKRKGSQKDLRPPDLWIHHEEMEMKNIEK-PSGTDPAG  1175
Hs_Neogenin    CTRRTTSHQKKKRAACKSVNGSHKYKGNSKDVKPPDLWIHHERLELKPIDK-SPDPNPIM  1185
Dm_Frazzled    CRRKPQSSPEHTKKSYQ--------KNNVGVPKPPDLWIHHDQMELKNIDKGLHTVTPVC  1300

Hs_DCC         RDSPIQS-CQDLTPVSHSQSETQLGSKSTSHSGQDTEEAGSSMSTLERSLAARRAPRAKL  1234
Hs_Neogenin    TDTPIPRNSQDITPVDNS-MDSNIHQRRNSYRGHESEDS---MS----TLAGRRGMRPKM  1237
Dm_Frazzled    SDGASSSGALTLP-------------RSVVHSEYEVETP----------------VPGHV  1331

Hs_DCC         MIPMDAQSNNPAVVSAIPVPTLESAQYPGILPSPTCGYPHPQFTLRP-VPFPT-LSVDRG  1292
Hs_Neogenin    MMPFDSQPPQP-VISAHPIHSLDNPHHH--FHSSSLASPARSHLYHPGSPWPIGTSMSLS  1294
Dm_Frazzled    TNSLDKRSYVPGYMTTSMNGTMERPQYP----RTQYSHQNRSHMTMEAGLSQQSLTQPQS  1387

                                                                |-------
Hs_DCC         FGAGRSQSVSEGPTTQQPPMLPP-------SQPE--------HSSSEEAPSRTIPTACVR  1337
Hs_Neogenin    DRANSTESVRNTPSTDTMPASSSQTCCTDHQDPEGATSSSYLASSQEEDSGQSLPTAHVR  1354
Dm_Frazzled    NSMAQTPEHPYGGYDANFCN---------------------AGNAAAGNGCVSTIESSK  1425

               -------- P2 ------------|
Hs_DCC         PTHPLRSFANPLLPPP-MSAIEPKVPYTPLLSQPGPTLPKTHVKTASLGLAGKARSPLLP  1396
Hs_Neogenin    PSHPLKSFAVPAIPPPGPPTYDPALPSTPLLSQQALNHHIHSVKTASIGTLGRSR-PPMP  1413
Dm_Frazzled    RGHPLKSFSVPGPPPT--GGATPVTKHTPAVTIRPQNQSPYKKPSFSAATPNRLQ-----  1478

                                          |-------- P3 --------|
Hs_DCC         VSVPTAPEVSEESHKPTEDSANVYEQDDLSEQMASLEGLMKQLNAITGSAF--        1447
Hs_Neogenin    VVVPSAPEV-QETTRMLEDSESSYEPDELTKEMAHLEGLMKDLNAITTA----        1461
Dm_Frazzled    -----GGGSVVHSTDEIQRLAPSTSTEELNQEMANLEGLMKDLSAITANEFEC        1526
```

Figure S2

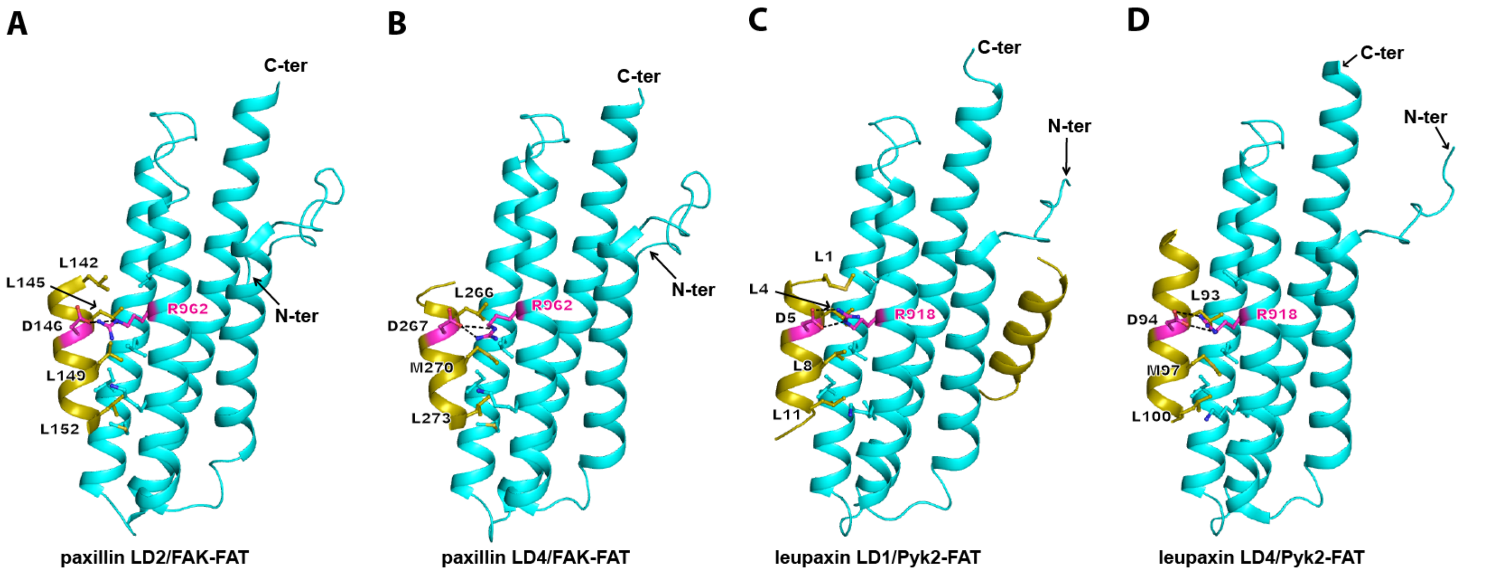
A
C-ter
N-ter
L142
L145
D146
R962
L149
L152
paxillin LD2/FAK-FAT
B
C-ter
N-ter
L266
D267
R962
M270
L273
paxillin LD4/FAK-FAT
C
C-ter
N-ter
L1
L4
D5
R918
L8
L11
leupaxin LD1/Pyk2-FAT
D
C-ter
N-ter
L93
D94
R918
M97
L100
leupaxin LD4/Pyk2-FAT

Figure S3

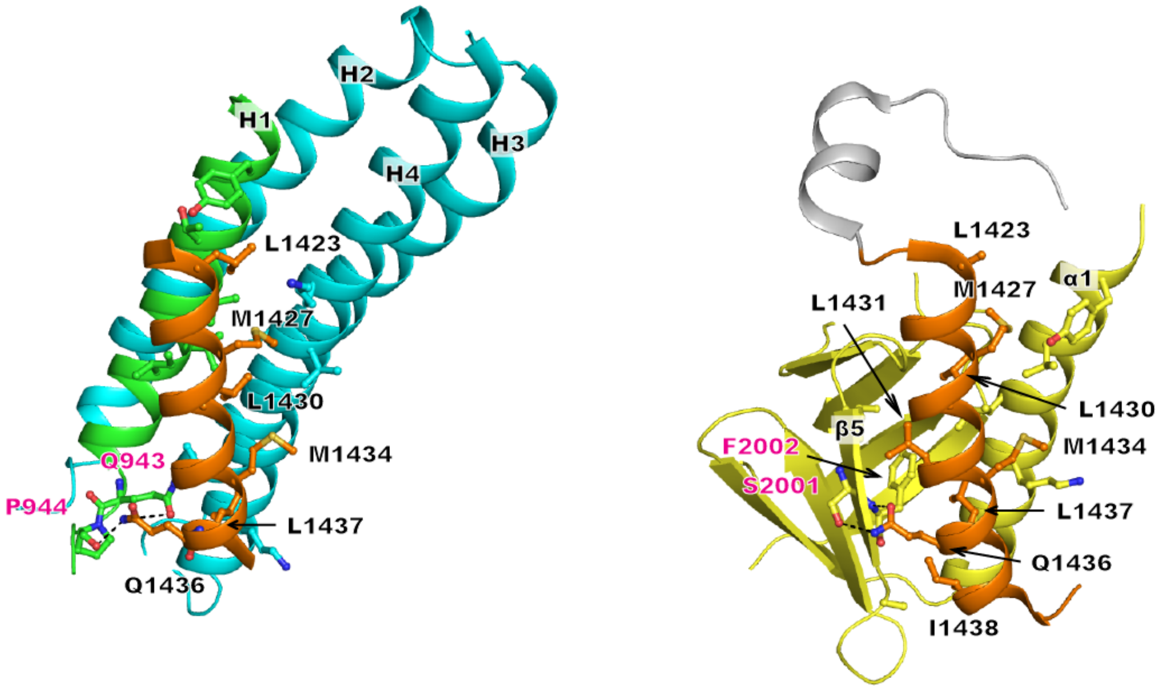

DCC-P3/FAK-FAT

DCC-P3/Myosin X MyTH4-FERM

Figure S4

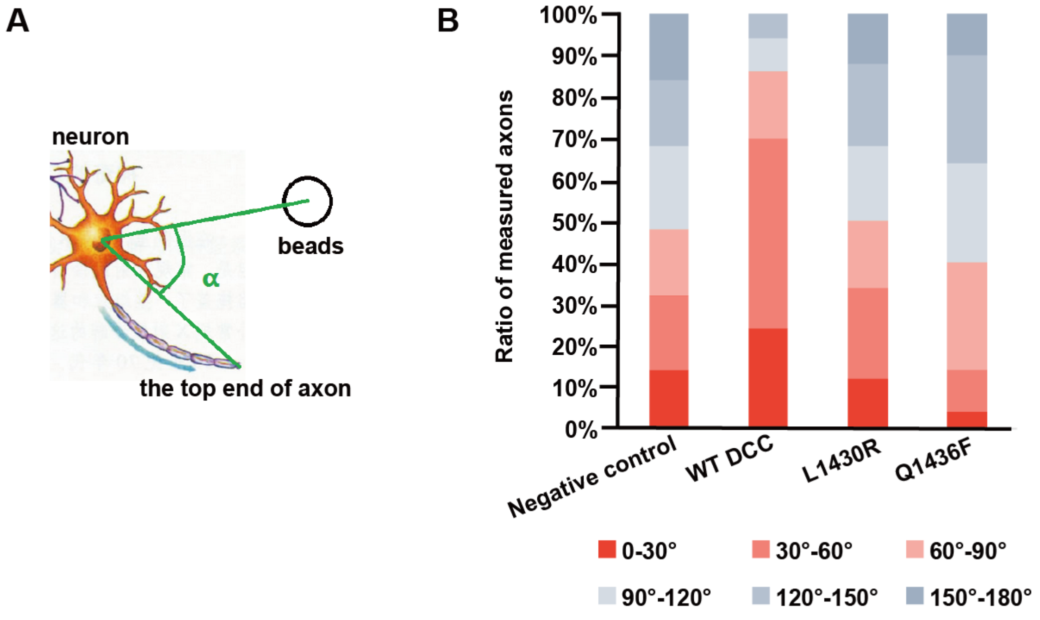
A
neuron
beads
α
the top end of axon
B
Ratio of measured axons
100%
90%
80%
70%
60%
50%
40%
30%
20%
10%
0%
Negative control
WT DCC
L1430R
Q1436F
0-30°
30°-60°
60°-90°
90°-120°
120°-150°
150°-180°

Figure S5

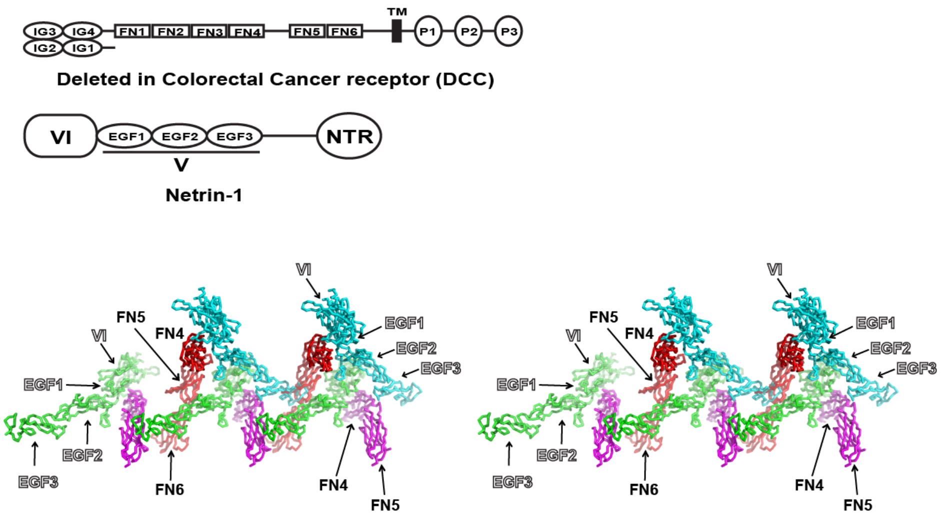
IG3
IG4
IG2
IG1
FN1
FN2
FN3
FN4
FN5
FN6
TM
P1
P2
P3
Deleted in Colorectal Cancer receptor (DCC)
VI
EGF1
EGF2
EGF3
V
NTR
Netrin-1
VI
FN5
FN4
EGF1
EGF2
EGF3
FN6

Supplemental Table S1. Statistics of the FAT-related structures in Protein Data Bank

	PDB code	Method	Resolution	Ligand	Dimer/Monomer	Brief description	Citation
FAK FAT	1K40	X-ray	2.25 Å		Monomer		[1]
	1K04	X-ray	1.95 Å		Dimer		[2]
	1K05	X-ray	2.9 Å		Monomer		
	1KTM	NMR			Monomer		[3]
	1OW6	X-ray	2.35 Å	Paxillin-LD4	Dimer/monomer	One FAT dimer with LD4 bound at H1/H4 interface, one FAT monomer with LD4 bound at H2-H3 interface, and another FAT monomer without ligand	[4]
	1OW7	X-ray	2.6 Å	Paxillin-LD4	Monomer	One with LD4 bound at H1/H4 interface and two with LD4 bound at H2/H3 interfaces	
	1OW8	X-ray	2.85 Å	Paxillin-LD2	Monomer	One with LD2 bound at H1/H4 interface, one with LD2 bound at H2/H3 interface, and one without ligand	
	1PV3	NMR			Monomer		[5]
	1QVX	NMR			Monomer		[6]
	2L6F	NMR		Paxillin-LD2/ Paxillin-LD4	Monomer	LD2 and LD4 were both artificially fused at the C-terminus of FAT. LD2 binds at H1/H4 interface and LD4 binds at H2/H3 interface.	
	2L6G	NMR		Paxillin-LD2	Monomer	LD2, which was fused at the C-terminus of FAT, binds at H1/H4 interface.	
	2L6H	NMR		Paxillin-LD4	Monomer	LD4, which was fused at the C-terminus of FAT, binds at H2/H3 interface.	
	3B71	X-ray	2.82 Å	CD4 endocytosis motif	Monomer	One with CD4 bound at H1/H4 interface and two with CD4 bound at H2/H3 interfaces	[7]
	3S9O	X-ray	2.6 Å		Monomer		[8]
Pyk2 FAT	3GM1	X-ray	2.95 Å	Paxillin-LD4	Monomer	LD4 motifs bind at H1/H4 and H2/H3 interfaces.	[9]
	3GM2	X-ray	2.71 Å		Monomer		
	3GM3	X-ray	2.6 Å		Monomer		
	2LK4	NMR			Monomer		[10]
	3U3F	X-ray	3.1 Å	Paxillin-LD2/ Paxillin-LD4	Monomer	Two with LD4 bound at H1/H4 interface and LD2 bound at H2/H3 interface, another two with LD2 bound at H2/H3 interface	
	4R32	X-ray	3.51 Å	Paxillin-LD2	Monomer	LD2 motifs bind at H1/H4 and H2/H3 interfaces.	
	4XEF	X-ray	2.5 Å	Leupaxin-LD1	Monomer	LD1 motifs bind at H1/H4 and H2/H3 interfaces.	[11]
	4XEK	X-ray	1.79 Å	Leupaxin-LD4	Monomer	LD4 binds at H2/H3 interface.	
	4XEV	X-ray	2.01 Å	Leupaxin-LD1/ Leupaxin-LD4	Monomer	LD1, which was fused at the C-terminus of FAT, binds at H1/H4 interface. LD4 binds at H2/H3 interface.	

Supplemental References

1. Hayashi, I, Vuori, K, and Liddington, RC. The focal adhesion targeting (FAT) region of focal adhesion kinase is a four-helix bundle that binds paxillin. *Nat Struct Biol* 2002; 9:101-106.
2. Arold, ST, Hoellerer, MK, and Noble, ME. The structural basis of localization and signaling by the focal adhesion targeting domain. *Structure* 2002; 10:319-327.
3. Liu, G, Guibao, CD, and Zheng, J. Structural insight into the mechanisms of targeting and signaling of focal adhesion kinase. *Mol Cell Biol* 2002; 22:2751-2760.
4. Hoellerer, MK, Noble, ME, Labesse, G, Campbell, ID, Werner, JM, and Arold, ST. Molecular recognition of paxillin LD motifs by the focal adhesion targeting domain. *Structure* 2003; 11:1207-1217.
5. Prutzman, KC, Gao, G, King, ML *et al.* The focal adhesion targeting domain of focal adhesion kinase contains a hinge region that modulates tyrosine 926 phosphorylation. *Structure* 2004; 12:881-891.
6. Gao, G, Prutzman, KC, King, ML *et al.* NMR solution structure of the focal adhesion targeting domain of focal adhesion kinase in complex with a paxillin LD peptide: evidence for a two-site binding model. *J Biol Chem* 2004; 279:8441-8451.
7. Garron, ML, Arthos, J, Guichou, JF, McNally, J, Cicala, C, and Arold, ST. Structural basis for the interaction between focal adhesion kinase and CD4. *J Mol Biol* 2008; 375:1320-1328.
8. Kadare, G, Gervasi, N, Brami-Cherrier, K *et al.* Conformational dynamics of the focal adhesion targeting domain control specific functions of focal adhesion kinase in cells. *J Biol Chem* 2015; 290:478-491.
9. Lulo, J, Yuzawa, S, and Schlessinger, J. Crystal structures of free and ligand-bound focal adhesion targeting domain of Pyk2. *Biochem Biophys Res Commun* 2009; 383:347-352.
10. Vanarotti, MS, Miller, DJ, Guibao, CD, Nourse, A, and Zheng, JJ. Structural and mechanistic insights into the interaction between Pyk2 and paxillin LD motifs. *J Mol Biol* 2014; 426:3985-4001.
11. Vanarotti, MS, Finkelstein, DB, Guibao, CD, Nourse, A, Miller, DJ, and Zheng, JJ. Structural Basis for the Interaction between Pyk2-FAT Domain and Leupaxin LD Repeats. *Biochemistry* 2016; 55:1332-1345.
